# Application of Ethanol Extracts From *Alnus sibirica* Fisch. ex Turcz in Hair Growth Promotion

**DOI:** 10.3389/fbioe.2021.673314

**Published:** 2021-06-08

**Authors:** Eun Ju Ha, Jang-Hyuk Yun, Chuanling Si, Young Soo Bae, Young-Hwan Jeong, Kwang-Hyun Park, Sun-Eun Choi

**Affiliations:** ^1^Department of Cosmetology Science, Nambu University, Gwangju, South Korea; ^2^College of Veterinary Medicine and Institute of Veterinary Science, Kangwon National University, Chuncheon, South Korea; ^3^Tianjin Key Laboratory of Pulp and Paper, Tianjin University of Science and Technology, Tianjin, China; ^4^Department of Forest Biomaterials Engineering, College of Forest and Environmental Sciences, Kangwon National University, Chuncheon, South Korea; ^5^Department of Emergency Medical Rescue, Nambu University, Gwangju, South Korea; ^6^Department of Emergency Medicine, Graduate School of Chonnam National University Gwangju, Gwangju, South Korea

**Keywords:** *Alnus sibirica*, oregonin, constituent, biomass, bio-application, hair growth promoting

## Abstract

*Alnus sibirica* Fisch. ex Turcz (ASFT), belonging to the family of Betulaceae, grows naturally in Asia, Europe, and America. The aims of this study are determining the efficacy of various biomarkers related to hair loss, evaluated by extracting the branch with 60% alcohol, and purely separating diarylheptanoid oregonin, an indicator and active substance, from 60% alcohol extract of the tree. To determine the preventive effects on hair loss, we investigated the anti-oxidative and anti-apoptotic effects on hydrogen peroxide-induced cytotoxicity on human hair dermal papilla cells using 3-(4,5-dimethylthiazol-2-yl)-2,5-diphenyltetrazolium bromide assay and Western blotting analysis for proving of apoptosis-related marker alteration, respectively. Moreover, we examined the ameliorative effects of 60% alcohol extract of the tree and oregonin against changes of oxidative stress-induced cytokine and testosterone-induced dihydrotestosterone production as crucial pathways of the hair loss mechanism. These results suggest that 60% alcohol extract of the tree and oregonin were available as novel natural materials for maintaining hair health in mammals.

## Introduction

*Alnus sibirica* Fisch. ex Turcz belongs to the family Betulaceae, and more than 17 species of plants of the same genus grow naturally in the Republic of Korea (Lee, 1996). It is a tree resource that grows wild throughout the northern hemisphere such as the Republic of Korea, the United States, Japan, and China (Lee, 1997).

Until now, we isolated diarylheptanoid compounds from plants of the genus *Alnus* and reported various physiological activities such as anti-oxidant activity, anti-inflammatory activity, and anti-atopic activity (Choi et al., 2010). Through these previous studies, we have confirmed the functionality and utility of resources of the genus *Alnus* (Choi et al., 2012). This potent and effective physiological active substance is thought to be oregonin, known as a diarylheptanoid family compound present in high content in plants of the genus *Alnus*. That can be estimated by examining domestic and international research trends focusing on oregonin as an indicator material in the study of chemical system classification of plants of the genus *Alnus* (Lim et al., 2004; Choi, 2013).

A number of studies have been conducted to determine the structure by separating oregonin, a glycoside of diarylheptanoid (Karchesy et al., 1974; Suga et al., 1982; Ohta et al., 1984; Aoki et al., 1990; Choi et al., 2012; Ko et al., 2015; Hu et al., 2018; Huang et al., 2021), by tracking substances that change color to reddish brown when trees of the genus *Alnus* (*Alnus rubura*, *Alnus hirsuta*, and *Alnus japonica*) (Asakawa, 1971; Karchesy et al., 1974) are cut. In addition, many researchers around the world have published a number of research reports using oregonin as an indicator material in the chemical system classification study of the genus *Alnus* (Lee et al., 1992; Guz et al., 2002; Lim et al., 2004; Choi, 2013; Choi et al., 2016).

Diarylheptanoids isolated from plants of the genus *Alnus* prevent oxidation, prevent diarrhea, have anti-cancer effects (Sheth et al., 1973; Kawai et al., 1990; Lee et al., 1998), and inhibit the proliferation and NO production of mouse melanoma cell line B16-F10. In addition, it has a cyclooxygenase-2 inhibitory effect (Lee et al., 2000a), has an effect on dermal inflammation by acting as a 5-lipoxygenase inhibitor, inhibits platelet aggregation, acts as PKC alpha inhibitor, inhibits the enzyme activity involved in prostaglandin biosynthesis, and has antibacterial (Saxena et al., 1995) and liver-protective effects. Recently, it was found that it has anti-atopic efficacy *in vitro* and *in vivo* (Choi et al., 2010). In addition, it was found that oregonin has anti-apoptosis modulation effect by confirming that human dermal papilla cell apoptosis induced by oxidative stress is blocked by oregonin isolated from *A. japonica* branches (Lee et al., 2018). Therefore, many researchers around the world are characteristically paying attention to oregonin among the diarylheptanoid family as the representative compound of the effective substance or indicator substance of the genus *Alnus* (Sin and Ahn, 1991; Yamazaki et al., 1998; Lee et al., 2000b). The *Alnus* plant species is colloquially referred to as *Jeok-Yang* among Chinese traditional doctors and Korean oriental medicine. It is believed that it “clears body heat” and “reduces body heat.” In Chinese and Korean traditional medicine, it has been believed that body heat was closely related with skin diseases (Yang et al., 1999). Accordingly, *Alnus* species has been used in America as a traditional medicine for various skin afflictions (Boericke, 1927). With regard to the application of *Alnus* plants species to various skin diseases in the East and the West, this study attempted to conduct scientific verification on the prevention and treatment of hair loss diseases, which are getting a lot of interest and demand worldwide in recent years.

In addition, the research team is conducting research by utilizing the know-how of extracting and manufacturing poly-phenol compounds in high content—in this case, an effective substance derived from arboretum, which has recently been found to have various positive physiological activity effects (Hu et al., 2017; Dong et al., 2020; Pei et al., 2020; Xu et al., 2021). As a result, by extracting oregonin, a glycoside of diarylheptanoid, from ASFT in high content (Choi, 2019), it has been found to have antioxidant, anti-inflammatory, and anti-allergic effects.

In this study, the efficacy of various biomarkers related to hair loss was evaluated by extracting the ASFT branch with 60% alcohol and singly isolating oregonin, an indicator and active substance, from the 60% alcohol extract of ASFT.

While the market demands related to hair loss, both domestic and in countries abroad, are rapidly increasing recently, it is not easy to discover new natural materials that are safe for the human body with scientific efficacy as collateral.

In this study, the research group confirmed the ability of human dermal papilla cells to inhibit dihydrotestosterone (DHT) production, which is well known as a representative factor in male pattern hair loss. Finally, efficacy experiments were conducted on biomarkers related to oxidative stress-induced apoptosis, namely, Bcl-2, Bax, caspase-3, and PARP.

By carrying out the above-mentioned experimental results, we tried to confirm the possibility of a natural new material related to hair loss with oregonin isolated therefrom.

Thus, we performed the experiments and suggestions of the patho-/pharmaco-physiological evidence of the species and oregonin, typical effective substances of ASFT, reporting its main mechanisms of action, as well as to critically analyze its performance in the fight against oxidative stress-induced hair papilla cell damages and hair loss.

## Materials and Methods

### Preparation of ASFT Extract and Isolation

ASFT was collected at 10 kg (branches with barks) from the Special Resource Test Forest (Jinae-ri, Sinbuk-eup, Chuncheon-si, Gangwon-do), Republic of Korea, in September of 2020, certified by Prof. Choi (Wood–Natural Product Functional Materials Lab, College of Forest and Environmental Sciences, Kangwon National University), and a voucher specimen (ASB2020-09) was deposited at the herbarium of the College of Forest and Environmental Sciences, Kangwon National University. The 10-kg ASB was extracted twice with 60% edible ethanol extract at room temperature. The concentration that is removed by the edible ethanol under vacuum afforded to 661 g (6.61%).

Prep-HPLC was used to isolate oregonin from the 60% ethanol extract of ASFT. Silica gel was used as the column, and chloroform/methanol/water (70:30:4) solution was applied as an isocratic condition. These conditions were repeated to obtain a final 100 mg of pure oregonin, which was used in the experiment.

### Oregonin

**FIGURE 1:**
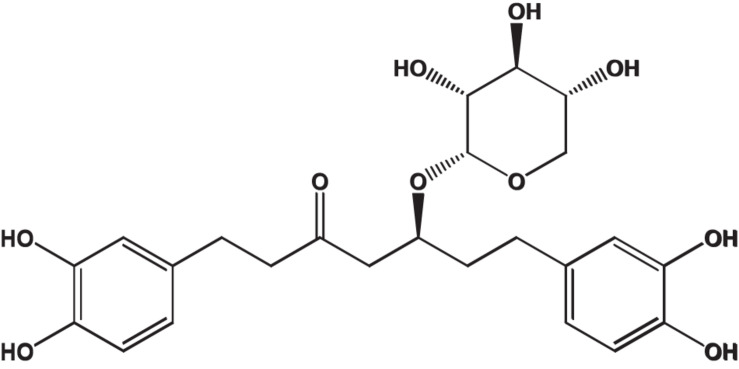
Chemical structure of oregonin. This figure was made with ChemDraw (http://www.perkinelmer.com/category/chemdraw) (Choi, 2019).

Oregonin, a brown amorphous powder, was purified and characterized following the previous reports (Choi, 2019) (positive LC/MS *m/z*: 501 [M + Na]^+^, negative LC/MS *m/z*: 477 [M - H]^–^). Definition of the chemical structure of purified oregonin (Figure 1) was made with ChemDraw (PerkinElmer, MA, United States).

### Cell Culture and Treatment

Human follicle dermal papilla cells (HFDPC) were obtained and cultured in the American Type Culture Collection (VA, United States), following the manufacturer’s instruction, in a 37°C humidified incubator supplied with 5% CO_2_ and 95% O_2_. The cells were seeded and were pretreated for 20 min with various concentrations of drugs and were subsequently incubated with hydrogen peroxide (H_2_O_2_, Sigma-Aldrich, MO, United States) for the indicated time points.

### MTT Assay

Cell viability was calculated by 3-(4,5-dimethylthiazol-2-yl)-2,5-diphenyltetrazolium bromide (MTT) assay with slight modifications (Si et al., 2013b; Hu et al., 2014, 2016). MTT (Sigma-Aldrich, MO, United States) was used as stock solution (×10, 5 mg/ml) with phosphate-buffered saline (PBS, pH 7.2) and filtered. At the end of the indicated treatment time, 10 μl of MTT solution was added to each well. After incubation for 20 min at 37°C, 100 μl of dimethyl sulfoxide was added to each well. After 20 min of incubation, the plate was read by a multiwell plate reader (BMG-biotech, Berlin, GmBh) at 570 nm for absorbance. The percentage of the live cells was calculated using the following formula: viability (%) = [(absorbance of sample)/(absorbance of control) × 100].

### Western Blot

Cell lysates were separated with 10 or 12% SDS-PAGE gels and transferred using 20% methanol onto polyvinylidene fluoride (PVDF) membranes (Bio-Rad laboratory, CA, United States). The membranes were blocked for 1 h at room temperature in 3% immunoglobulin-free bovine serum albumin (Invitrogen, CA, United States)-Tris buffered saline (pH 7.4). Primary antibodies to each antigen were administrated with their respective blocking buffers, following the manufacturer’s instruction, overnight at 4°C under gentle shaking. Washes were performed with PBS 0.1% Tween-20 (PBST) before the addition of secondary antibody for 1 h at room temperature. Washes were performed with 1 × PBST before imaging on chemoluminescence dye (Bio-Rad Laboratory, CA, United States). Protein detection was performed using Image Quent^TM^ with LAS-500 (GE Healthcare, CA, United States).

### ELISA

To measure insulin-like growth factor 1 (IGF-1) (Elh-IGF1, RayBiotech, GA, United States), transforming growth factor-β1 (TGF- β1, Enzo Life Science Inc, NY, United States), and dihydrotestosterone (ALPCO, NH, United States) levels in the cell lysate, enzyme-linked immunosorbent assay was used. All reaction and results were calculated after data were obtained with a microplate reader (BMG Biotech, Berlin, GmBH) at a suggested wavelength according to the manufacturer’s instruction.

### Statistical Analysis

Student’s *t*-test was conducted to assess the effects of the materials on superoxide-induced HFDPCs’ damage mechanism. Data are presented as mean ± standard deviation of the mean and as statistical comparisons between each group. Data are representative of three independent experiments. Significance was set at *p* < 0.05.

## Results

### Reduction of Oxidative Stress-Induced Cytotoxicity by Treatment With 60% ASFT EtOH Extracts and Oregonin

**FIGURE 2:**
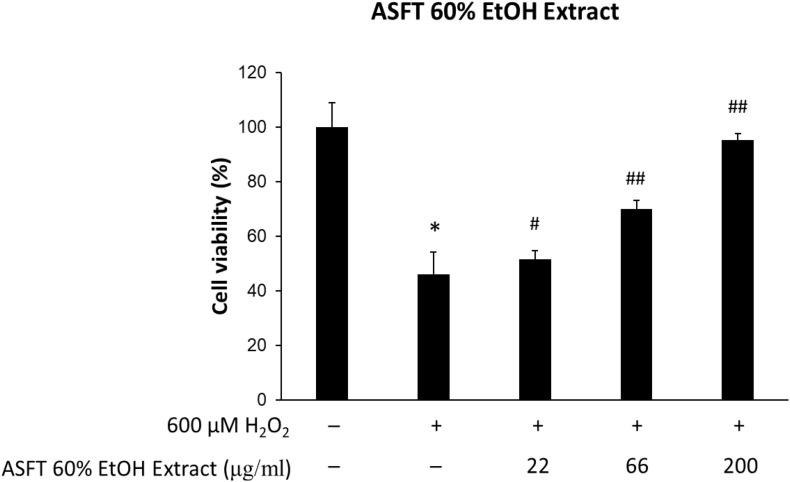
Inhibitory effects of 60% ethanol extract from *Alnus sibirica* Fisch. ex Turcz (ASFT) on oxidative stress in cells. The cells were pre-incubated with the indicated dose of ASFT (μg/ml, w/v) and treated with 600 μM H_2_O_2_. Cell viability was calculated as described in “Materials and Methods.” Data were expressed as mean ± SD. *P < 0.05 *vs* normal group. ^#^*P* < 0.05 and ^##^*P* < 0.01 vs H_2_O_2_-treated group. +, present; –, absent.

**FIGURE 3:**
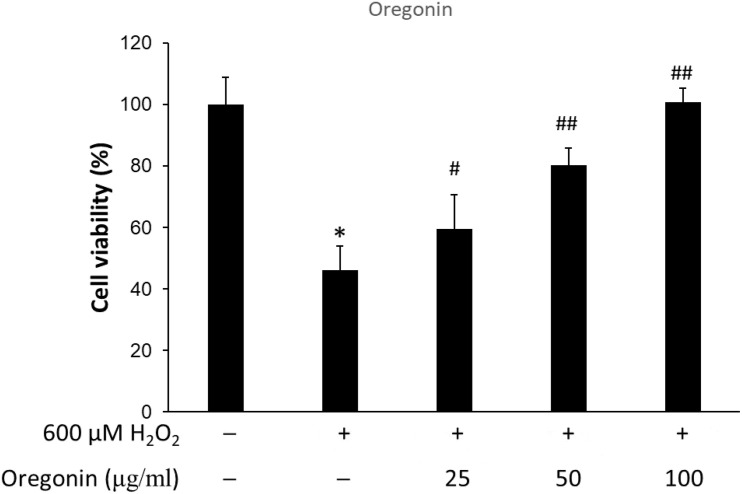
Inhibitory effects of oregonin on oxidative stress in cells. The cells were pre-incubated with the indicated dose of oregonin (μg/ml, w/v) and treated with 600 μM H_2_O_2_. Cell viability was calculated as described in “Materials and Methods.” Data were expressed as mean ± SD. *P < 0.05 *vs* normal group. ^#^*P* < 0.05 and ^##^*P* < 0.01 *vs* H_2_O_2_-treated group. +, present; –, absent.

The protective effects of 60% EtOH extracts from ASFT and oregonin on oxidative stress-induced HDFPC were evaluated by MTT assay. The results showed that 60% EtOH extracts from ASFT had inhibitory effects on 600 μM H_2_O_2_-induced cytotoxicity (Figure 2). The viability of HDFPC was reduced by 600 μM H_2_O_2_ to 50%, and the 60% EtOH extracts from ASFT were gradually increased in viability in a dose-dependent manner. Similarly, oregonin also had ameliorative effects on the same damage model in HDFPC (Figure 3). Isolated oregonin significantly protected against oxidative stress-induced cytotoxicity with a smaller dose than the ethanol extracts.

### Protective Mechanisms Against Oxidative Stress-Induced Apoptosis by 60% ASFT EtOH Extracts and Oregonin

**FIGURE 4:**
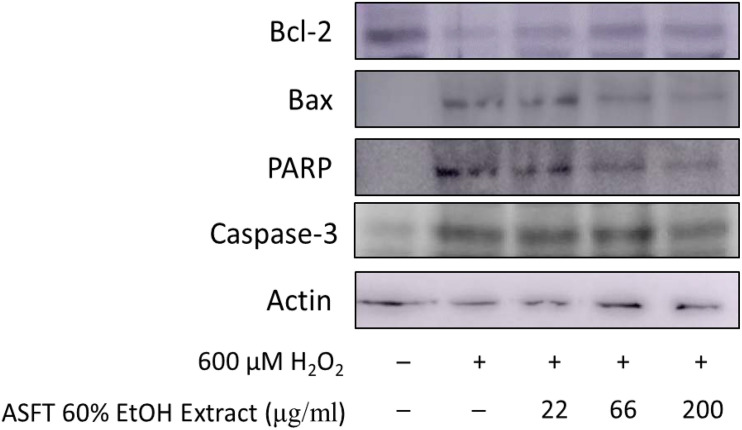
Anti-apoptotic effects of 60% ethanol extract from *Alnus sibirica* Fisch. ex Turcz (ASFT) on H_2_O_2_-induced oxidative damage in hair follicle dermal papilla cells. The cells (1 × 10^5^ cells) were pre-incubated with 60% ethanol extract from ASFT in various dosages or in control medium for 10 min at 37°C in a CO_2_ incubator. Then, the cells were treated with H_2_O_2_ (final concentration, 600 μM) and further incubated for 12 h. The harvested cells were solubilized with protease inhibitors (Sigma-Aldrich, MO, United States) containing radioimmunoprecipitation assay buffer (Elpis Biotech, Seoul, South Korea) and analyzed by western blotting by using PARP-1, Bax, Bcl-2, caspase-3, and β-actin-specific antibody. +, present; –, absent.

**FIGURE 5:**
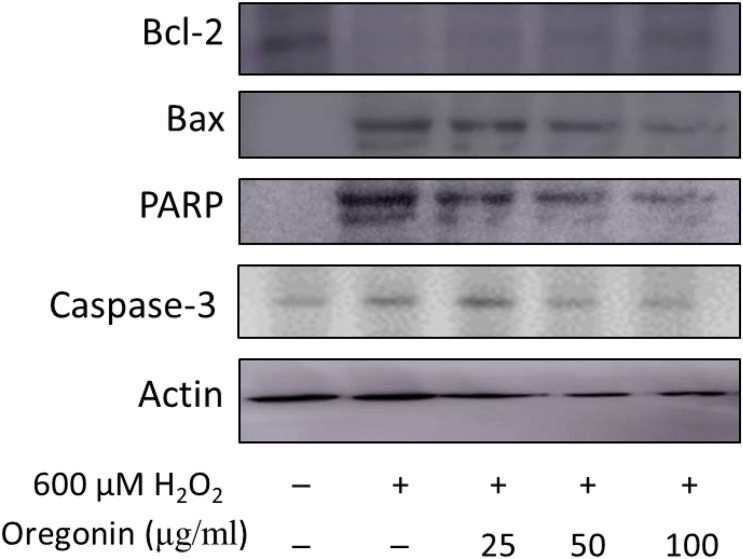
Anti-apoptotic effects of oregonin on H_2_O_2_-induced oxidative damages in hair follicle dermal papilla cells. The cells (1 × 10^5^ cells) were pre-incubated with oregonin in various dosages or in control medium for 10 min at 37°C in a CO_2_ incubator. Then, the cells were treated with H_2_O_2_ (final concentration, 600 μM) and further incubated for 12 h. The harvested cells were solubilized with protease inhibitors (Sigma-Aldrich, MO, United States) containing radioimmunoprecipitation assay buffer (Elpis Biotech, Seoul, South Korea) and analyzed by western blotting by using PARP-1, Bax, Bcl-2, caspase-3, and β-actin-specific antibody. Data were expressed as representative results from three independent experiments. +, present; –, absent.

In the same manner as described above, we performed experiments wherein 600 μM of H_2_O_2_ was treated to measure apoptosis in HDFDC. To elucidate the protective mechanisms of 60% EtOH extracts from ASFT and oregonin on oxidative stress-induced HDFPC apoptosis, we analyzed apoptosis marker molecules including Bcl-2 (representative protein molecule involved in inhibition of apoptosis) (Tsujimoto et al., 1985; Cleary et al., 1986) and Bax (Bcl-2-associated X protein, representative apoptosis-inducing protein molecule) (Oltvai et al., 1993; Sedlak et al., 1995), poly ADP-ribosyl polymerase 1 (PARP-1), and caspase-1 (Figures 4, 5). Treatment with H_2_O_2_ significantly reduced the expression of Bcl-2 molecules, while pretreatment with 60% EtOH extracts from ASFT (Figure 4) and oregonin (Figure 5) markedly elevated the expression of Bcl-2 molecules in a dose-dependent manner. In contrast, treatment with H_2_O_2_ elevated the expression of Bax, PARP, and caspase-1 protein level. The pretreatment with 60% EtOH extracts from ASFT (Figure 4) and oregonin (Figure 5) showed reducing properties to these apoptosis signals in a dose-dependent manner.

### Efficacy in Increasing IGF-1 Expression

**FIGURE 6:**
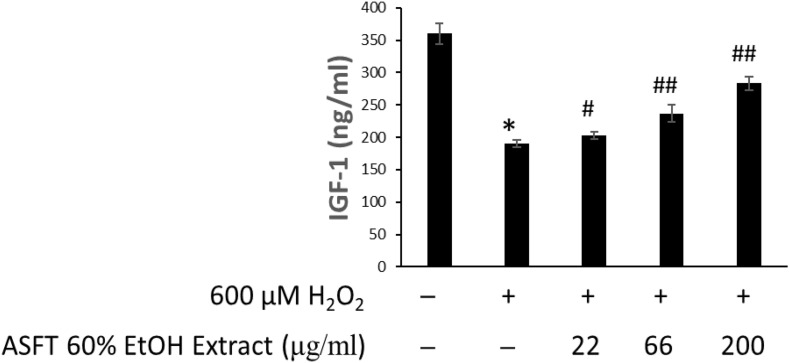
Increasing effect of 60% ethanol extract from *Alnus sibirica* Fisch. ex Turcz (ASFT) on IGF-1 formation. The cells were pre-incubated with the indicated dose of 60% ethanol extract from ASFT and treated with H_2_O_2_. IGF-1 formation was measured as described in “Materials and Methods” following the manufacturer’s instructions. Data were expressed as mean ± SD. *P < 0.01 *vs* normal group. ^#^*P* < 0.05 and ^##^*P* < 0.01 *vs* H_2_O_2_-treated group. Data were expressed as representative results from three independent experiments. +, present; –, absent.

**FIGURE 7:**
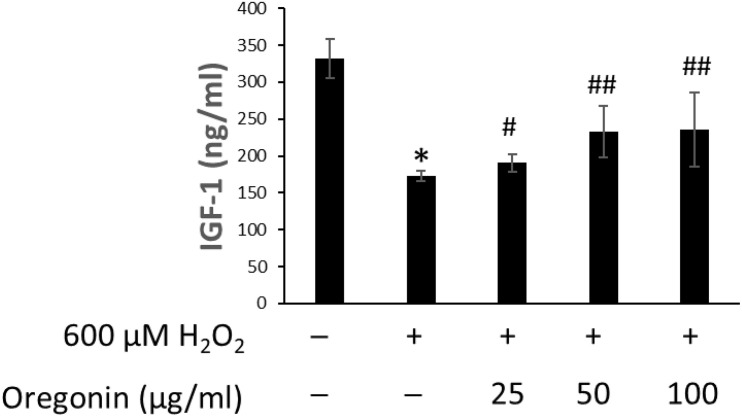
Increasing effect of oregonin on IGF-1 formation. The cells were pre-incubated with the indicated dose of oregonin and treated with H_2_O_2_. The IGF-1 content (ng/ml) in the cell lysates was measured as described in the manufacturer’s instruction. Data were expressed as mean ± SD. *P < 0.01 *vs* normal group. ^#^*P* < 0.05 and ^##^*P* < 0.01 *vs* H_2_O_2_-treated group. +, present; –, absent.

The changes of IGF-1 level on oxidative stress-induced HDFPC damage and the recovery effects of 60% EtOH extracts from ASFT and oregonin are shown in Figures 6, 7, respectively. Treatment with H_2_O_2_ significantly decreased the IGF-1 level in the intracellular region, whereas 60% EtOH extracts from ASFT (Figure 6) and oregonin (Figure 7) treatment attenuated the oxidative stress-induced IGF-1 level in a dose-dependent manner compared to H_2_O_2_ alone.

### TGF-β1 Expression Inhibition Efficacy

**FIGURE 8:**
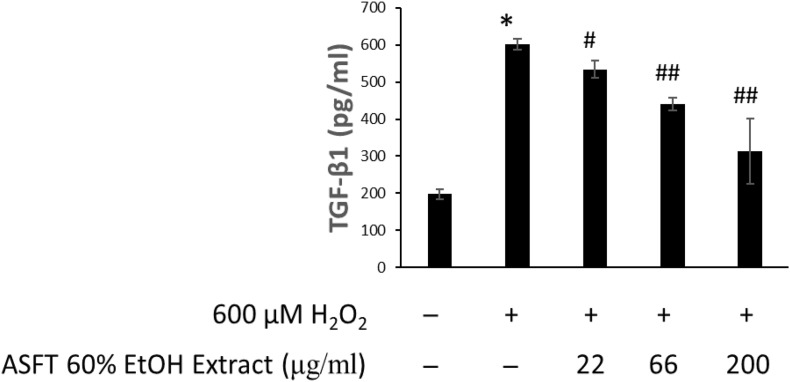
Inhibitory effect of 60% ethanol extract from ASFT on TGF-β formation. The cells were pre-incubated with the indicated dose of 60% ethanol extract from ASFT and treated with H_2_O_2_. TGF-β formation was measured as described in “Materials and Methods.” Data were expressed as mean ± SD. *P < 0.001 *vs* normal group. ^#^*P* < 0.05 and ^##^*P* < 0.01 *vs* H_2_O_2_-treated group. +, present; –, absent.

**FIGURE 9:**
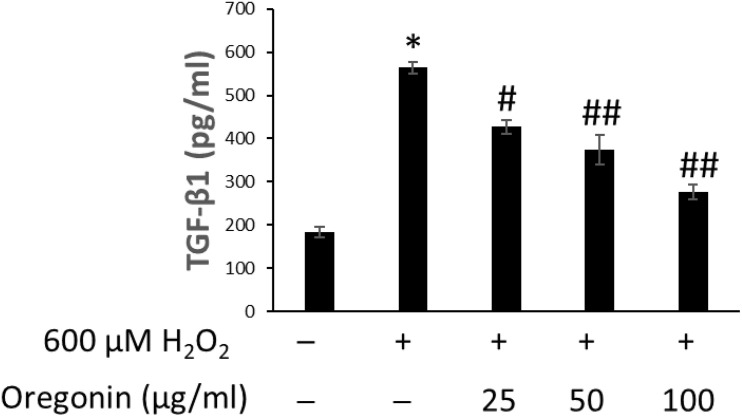
Inhibitory effect of oregonin on TGF-β formation. The cells were pre-incubated with the indicated dose of oregonin and treated with H_2_O_2_. The TGF-β content (pg/ml) in the cell lysates was measured as described in the manufacturer’s instruction. Data were expressed as mean ± SD. *P < 0.001 *vs* normal group. ^#^*P* < 0.05 and ^##^*P* < 0.01 *vs* H_2_O_2_-treated group. +, present; –, absent.

The effects of 60% EtOH extracts from ASFT and oregonin on TGF-β1 expression in the intracellular region were determined (Figures 8, 9, respectively). H_2_O_2_ treatment significantly increased TGF-β1 expression compared to the normal controls. However, 60% EtOH extracts from ASFT (Figure 8) and oregonin (Figure 9) treatment abrogated these effects in a dose-dependent manner, resulting in a significant reduction in TGF-β1 expression compared to H_2_O_2_ treatment alone.

### Dihydrotestosterone Production Inhibitory Effect

**FIGURE 10:**
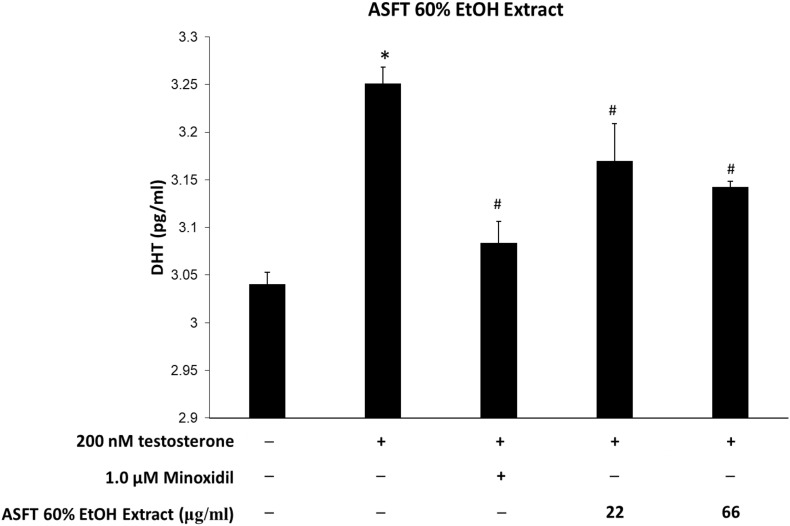
Inhibitory effect of 60% ethanol extract from *Alnus sibirica* Fisch. ex Turcz (ASFT) on dihydrotestosterone (DHT) formation. Cells were pre-incubated with the indicated dose of 60% ethanol extract from ASFT and treated with testosterone. The DHT content (pg/ml) in the cell lysates was measured as described in the manufacturer’s instruction. Data were expressed as mean ± SD. *P < 0.05 *vs* normal group. ^#^*P* < 0.05 *vs* testosterone-treated group. +, present; –, absent.

**FIGURE 11:**
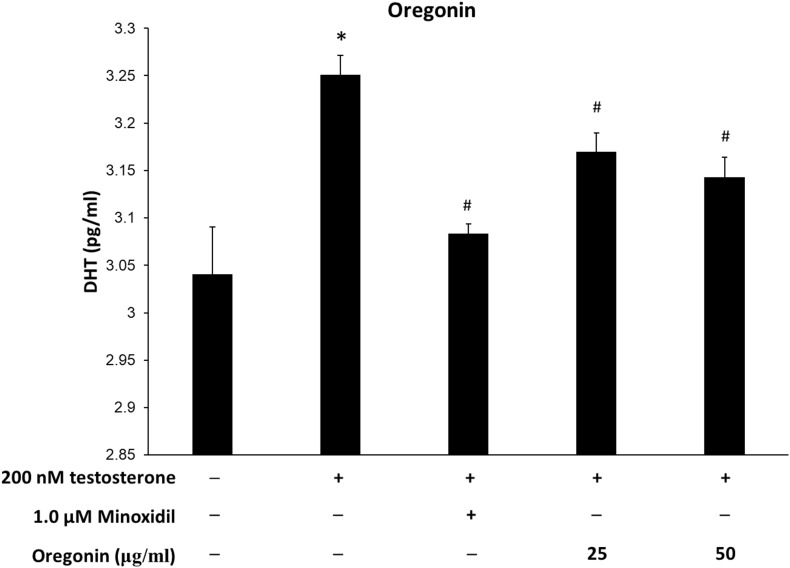
Inhibitory effect of oregonin on dihydrotestosterone (DHT) formation. The cells were pre-incubated with the indicated dose of oregonin and treated with testosterone. The DHT content (pg/ml) in the cell lysates was measured as described in the manufacturer’s instruction. Data were expressed as mean ± SD. *P < 0.05 *vs* normal group. ^#^*P* < 0.05 *vs* testosterone-treated group. +, present; –, absent.

Levels of DHT are shown in Figures 10, 11. The group treated with testosterone, the substrates of 5α-reductase, showed significantly higher levels of DHT than the normal control group. Simultaneous treatment with 60% EtOH extracts from ASFT (Figure 10) and oregonin (Figure 11) to testosterone-treated HDFPC significantly decreased the DHT content compared to testosterone treatment alone. The administration of minoxidil, a well-known inhibitor of 5α-reductase, also significantly reduced DHT production compared to the normal controls. Interestingly, 60% EtOH extracts from ASFT and oregonin showed a similar efficacy in a dose-dependent manner, respectively. These results indicate that 60% EtOH extracts from ASFT and oregonin had an ameliorative effect on DHT-mediated hair loss.

## Discussion

**FIGURE 12:**
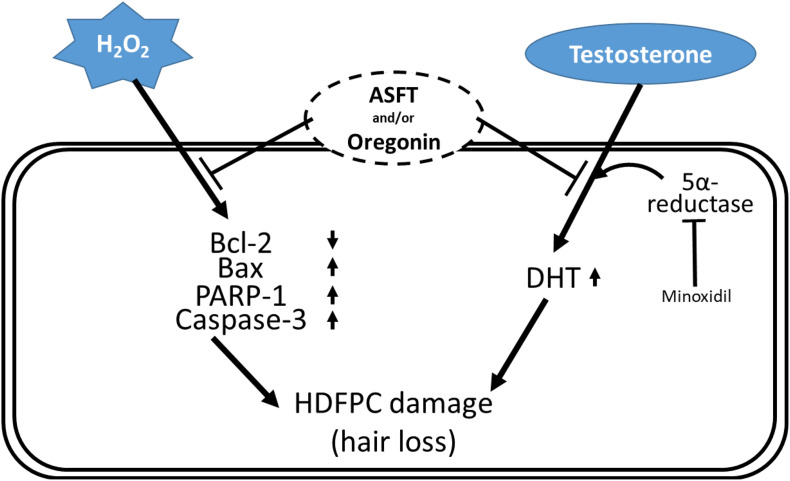
Schematic diagram for the hypothetical action mechanisms of 60% ethanol extract from *Alnus sibirica* Fisch. ex Turcz and oregonin on oxidative stress-induced hair follicle dermal papilla cell damages and dihydrotestosterone-mediated hair loss.

Different components in plants are a rich source far from being well utilized (Si et al., 2008b, 2009a; An et al., 2019; Du et al., 2019; Li et al., 2019; Liu et al., 2020a; Liu H. et al., 2021; Liu K. et al., 2021), and chemical constituents from plants demonstrate various significant bioactivities (Si et al., 2008a, 2009b, 2013a; Huang et al., 2018, 2019; Liu et al., 2020b). The present study indicates that 60% EtOH extracts from ASFT or oregonin treatment prevents oxidative stress-induced apoptosis and ameliorated DHT-mediated hair loss signaling (Figure 12) in different dose ranges between each material. The general pattern of male hair loss is determined by genetic causes, and when hair loss begins earlier, the degree of hair loss tends to become more severe. Hair loss symptoms are reported, such as the case that if at least one of the paternal or maternal family members has alopecia, then it is more likely to develop specifically in males in later generations (Olsen, 2001; Yip et al., 2011; Liu et al., 2017; Lu et al., 2019; Kumar et al., 2020).

Oxidative stress is known to induce apoptosis and/or stimulate various cell types within the scalp and hair components; these cell types are keratinocytes, hair follicle cells, papilla cells, and various immune cells. Programmed cell death, commonly referred to as apoptosis, is mediated by various signal cascades including Bax, Bcl-2, PARP-1, and caspase family. PARP-1 is present in the nucleus of cells and uses a large amount of NAD to induce poly-ADP ribosylation of the target protein. Through this, it activates signaling involved in sub-cell death. Activation of PARP-1 has been used as an indicator for the progression of apoptosis (Schultz et al., 2003; Godon et al., 2008). HDFDC treated with 600 μM of H_2_O_2_ significantly increased the expression of PARP-1 in cells, and this result proves that apoptosis of cells is in progress. An additionally identified biomarker is caspase-3. Caspase-3 is a molecule which is responsible for the final stage of apoptosis of cells and is a protein which is responsible for irreversible cell death through the expression of the above-mentioned various molecules and cell signaling mechanisms. Furthermore, oxidative stress can simulate with hydrogen peroxide, freely translocating to intra-/extra-cellular region. In many experimental trials, it serves as an inducer in normal physiology or malfunctions. As a result, the identification of caspase-3 activation or increased protein expression has been used as an important final indicator for the progression of apoptosis (Alnemri et al., 1996; Harrington et al., 2008). HDFDC treated with 600 μM of H_2_O_2_ significantly increased the protein expression of caspase-3 in the cells, and these results could confirm that the apoptosis of cells is actually in progress. In this study, 60% EtOH extracts from ASFT show different dose ranges larger than oregonin in most experiments, but ASFT contains more varied compounds. Thus, we found effective and safe dosages of ASFT and oregonin for these experimental models.

Growth factors in hair growth, e.g., IGF-1, have been reported as important growth factors involved in hair growth and regulation by promoting the growth of epithelial cells in culture, increasing the tissue length of hair follicles, and acting as an important factor promoting hair growth (Itami et al., 1995; Hibino and Nishiyama, 2004). IGF-1 is involved in the increased expression by androgenic hormones and the action of testosterone on the hair, and it is known to play an important role in the mechanism of action that causes androgen-dependent alopecia (Price, 1999; Randall, 2008).

In addition, it has been reported to not only prevent aging, improve dementia, depression, and adult lifestyle diseases but also enhance immunity and improve bone density, prevent skin aging, and induce the transformation of hair from a resting state to a growing state (Rosen et al., 1994). Research results on the increase or decrease in hair loss treatment have also been reported. Furthermore, as the growth of hair has a closer relationship with blood circulation, IGF-1 and EGF have a reported function that contributes to the growth of hair follicles that promote the growth of hair (Hodak et al., 1996; Wells, 1999). As such, increasing the expression of IGF-1 factor, which is an important factor in hair growth, can be expected to be effective in hair growth. Discovering the advantages of developing new materials derived from natural products that minimize the risk of side effects and toxicity of existing synthetic-based compounds is an important factor in developing new technologies. Therefore, we confirmed the effect of promoting the expression of IGF-1 growth factor in a concentration-dependent manner by treating dermal papilla cells with 60% alcohol extract and oregonin. These results are expected in such a way that both compounds will have a protective effect on hair loss, and the possibility of it being developed as a natural new material for hair health is suggested. Moreover, reactive oxygen species (ROS) formation was increased after the administration of androgens, and TGF-β1 secretion was increased by the administration of androgen in DP-6 cell line, whereas androgen-induced TGF-β1 was significantly inhibited by N-acetyl cysteine. In addition, the potential for the treatment of androgen-dependent alopecia has been reported by the induction of TGF-β1 by androgens by treatment with antioxidants to block the ROS signaling pathway of hair follicle cells (Olsen et al., 2006). In this study, we explored the 60% EtOH extract from ASFT and oregonin on IGF and TGF-β1 expression in HFDPC, which is helpful for hair loss. It shows their possibility as an excellent natural new material that can be used in hair loss prevention.

On the other hand, one of the representative factors which cause hair thinning or falling out is testosterone, a male hormone which is changed to dihydrotestosterone (DHT) by 5ɑ-reductase, and it causes the shrinkage of the dermal papilla on the scalp in people who are not fond of DHT. It is generated, and eventually final hair loss proceeds (Mounsey and Reed, 2009). Male hormones promote the positive function of masculinity and cause seborrheic dandruff to worsen the symptoms of alopecia (Ramos and Miot, 2015). The male hormone, which is the main cause of male pattern hair loss, causes hair loss not due to the amount of male hormone secretion in the hair but due to the influence of 5α-reductase, an active enzyme acting on male hormones. The changed DHT acts on the hair cells, slowing the atrophy and cell division of the hair follicles and resulting in softening hair and hair loss (Shapiro and Price, 1998). Minoxidil, a treatment for the positive control group used in this experiment, was developed as a vasodilator for the treatment of hypertension in the early 1970s but was used as a hair growth promoter when hirsutism was reported as a side effect (Zappacosta, 1980). The mechanism for the hair growth effect has not been accurately identified, and it has been reported that hair growth is induced by the increased nutritional supply due to vasodilation (Lachgar et al., 1998), but recently the media revealed the mechanism that minoxidil was effective in suppressing male pattern hair loss through the suppression of DHT production, and accordingly, more aggressive marketing promotions from related pharmaceutical companies are being made. Minoxidil is reported to be most effective only for initial hair loss, in which the hair partially falls out, and for the crown of ongoing hair loss. So only when it is used in the early stages of male pattern hair loss can it prevent the progression of hair loss and contribute to hair growth. It is said that there is no effect on the front of the scalp, which has become bald, and if it is not applied to the scalp steadily twice a day for 6 to 12 months, it is said that no effect is seen (Kurbel et al., 1999).

In the experiment, minoxidil was applied to the hair loss area; hair growth was promoted not only in men but also in women (Messenger and Rundegren, 2004), but when 5% minoxidil was used, the color of the cilia became darker and thicker, resulting in hirsutism. Therefore, products are manufactured and sold at low concentrations to women in Korea. The side effects include salt and moisture retention, swelling, tachycardia, local peeling, dermatitis, skin irritation such as soreness, erythema, and peeling at the site of administration (Suchonwanit et al., 2019) or itching, and dryness of the skin due to a small amount of the drug absorbed on the skin. Drops, redness, and contact dermatitis have been reported, and hirsutism can be induced in 0.5–1% (Peluso et al., 1997).

There may be a phenomenon in which the hair loss becomes worse due to the increase of telogen hair shedding due to the temporary effect of minoxidil on the hair cycle (Suchonwanit et al., 2019), Minoxidil’s hair growth promotion effect depends on whether or not hair growth is administered, and there is a shortcoming in the period of returning to the state before treatment when it is stopped (Zins, 1988). Due to concerns about these side effects, in recent years, the social demand for the development of new anti-hair loss materials derived from natural products, whose efficacy is scientifically confirmed while being safe for the human body, is high.

The series of the largest characteristic compounds in the *Alnus* species plant is the diarylheptanoid compound (Karchesy et al., 1974; Lee et al., 1992; Guz et al., 2002). Many researchers around the world, including this research team, have found that the representative indicator or effective compound of the *Alnus* species plant is oregonin (Ohta et al., 1984; Aoki et al., 1990; Choi et al., 2012; Ko et al., 2015), which is in the diarylheptanoid series (Asakawa, 1971; Karchesy et al., 1974; Suga et al., 1982; Lim et al., 2004; Choi, 2013). Therefore, in this study, we conducted various experiments related to improving and preventing hair loss by the separation and identification of the diarylheptanoid series, which is an indicator and effective substance from ASFT. Through this research, we believe that the bio-industry has recently conducted a research that suggests significance in the functional material market related to hair loss, which is a large global market, whereas the development of novel materials for prevention of hair loss and/or stimulation of hair growth requires animal experiments or human research for clinical use, but not the cosmetic area because of global ethics. Therefore, each of these materials need further experiments with simulation in human or animals for clinical purposes.

Accordingly, in this study, the result of conducting an experiment on the inhibition of dihydrotestosterone production, which is known as the main cause of male pattern hair loss, with 60% alcohol extract of ASFT and oregonin was shown to be statistically significant when compared with the positive and negative controls. By confirming the DHT reduction effect, 60% alcohol extract of ASFT and oregonin were confirmed to be effective anti-hair loss agents and new materials that promote hair growth for male pattern hair loss.

## Conclusion

As a product related to “hair loss symptom relief,” which was recently classified as a quasi-drug by the Ministry of Food and Drug Safety, this was included in the category of functional cosmetics, Therefore, it can be said to be one of great interest in related industries.

In this study, with the aim to discover new materials derived from natural products that can provide an objective basis for scientifically controlling hair loss while having a low risk of side effects, a limitation of existing drugs, it was confirmed that oregonin, isolated from the branches including the bark of ASFT, clearly regulates the death of dermal papilla cells, which is highly related to hair loss, based on strong antioxidant activity. In conclusion, it was confirmed that the anti-hair loss effect can be expected through the inhibition and control of the death of dermal papilla cells caused by oxidative stress based on the effect of powerfully removing oxidative stress from the scalp with ASFT extract and oregonin. In addition, when DHT, a representative causative agent of male pattern hair loss, was compared with the positive control, minoxidil, it was statistically and significantly reduced. It was also confirmed that IGF-1, a representative biomarker for promoting hair growth, was statistically and significantly increased and that TGF-β1, a representative biomarker for promoting hair loss, was statistically significant.

When the above-mentioned results are combined, it suggests the possibility of the development of a new natural material for hair growth to prevent male pattern alopecia and various hair loss symptoms caused by the death of dermal papilla cells (e.g., alopecia areata, stress-related hair loss, *etc*.).

## Data Availability Statement

The original contributions presented in the study are included in the article/supplementary material, further inquiries can be directed to the corresponding author/s.

## Author Contributions

EH and J-HY performed the investigation. K-HP and S-EC supervised the study. S-EC and K-HP contributed to writing—original draft. CS, Y-HJ, and YB contributed to writing—review and editing. All authors contributed to the article and approved the submitted version.

## Conflict of Interest

The authors declare that the research was conducted in the absence of any commercial or financial relationships that could be construed as a potential conflict of interest.

## Abbreviations

ASFT: *Alnus sibirica* Fisch. ex Turcz
DHT: dihydrotestosterone
MTT: 3-(4,5-dimethylthiazol-2-yl)-2,5-diphenyltetrazolium bromide
HFDPC: human follicle dermal papilla cells
ELISA: enzyme-linked immunosorbent assay
IGF-I: insulin-like growth factor-I
TGF- β: transforming growth factor-beta
Bax: Bcl-2-associated X protein
Bcl-2: B-cell lymphoma 2
PARP-1: poly ADP-ribose polymerase 1.
